# Electrochemical Immunosensors on Laser-Induced Graphene Platforms for Monitoring of Anti-RBD Antibodies After SARS-CoV-2 Infection

**DOI:** 10.3390/bios14110514

**Published:** 2024-10-22

**Authors:** Beatriz R. Martins, Cristhianne Molinero R. Andrade, Guilherme F. Simão, Rhéltheer de Paula Martins, Lucas V. de Faria, Tiago A. Matias, Virmondes Rodrigues Júnior, Rodrigo Alejandro Abarza Munoz, Renata Pereira Alves

**Affiliations:** 1 Department of Immunology, Federal University of Triângulo Mineiro, Uberaba 38025-180, Brazil; biaroma_95@hotmail.com (B.R.M.); cristhianne_m@hotmail.com (C.M.R.A.); guilhermefelipesimao@gmail.com (G.F.S.); rheltheer.martins@uftm.edu.br (R.d.P.M.); virmondes.rodrigues@uftm.edu.br (V.R.J.); 2 INCT-Neuroimmune Modulation, Uberaba 38025-350, Brazil; 3 Institute of Chemitry, Federal University of Uberlândia, Uberlândia 38408-100, Brazil; viniciuslucas82@yahoo.com.br (L.V.d.F.); tiago.matias@ufes.br (T.A.M.); 4 Institute of Agricultural, Exact and Biological Sciences, Biological Sciences Department, Federal University of Triângulo Mineiro, Iturama 38280-000, Brazil

**Keywords:** COVID-19, electrochemical biosensor, Point-of-Care Testing, immunoglobulins

## Abstract

The COVID-19 pandemic, caused by the SARS-CoV-2 virus, has posed a major challenge to global health. The development of fast, accurate, and accessible diagnostic methods is essential in controlling the disease and mitigating its impacts. In this context, electrochemical biosensors present themselves as promising tools for the efficient monitoring of SARS-CoV-2 infection. We have developed a highly specific biosensor for the detection of anti-SARS-CoV-2 antibodies in patient sera. The use of the RBD-S region as an antigen, although purified to minimize cross-linking, poses a specific challenge. The structural similarity between SARS-CoV-2 and other respiratory viruses, as well as the complexity of the serum matrix, hinders robust analytical strategies to ensure diagnostic accuracy. This work presents a novel immunosensor for COVID-19 diagnosis using laser-induced graphene (LIG) electrodes subjected to electrochemical reduction with graphene (named rGraphene-LIG). In the present study, we chose an initial approach focused on demonstrating the concept and evaluating the feasibility of the rGraphene-LIG sensor for SARS-CoV-2 detection. The rGraphene-LIG electrodes presented a notable current increase for the redox probe in the aqueous solution of a mixture of 5 mmol L^−1^ potassium ferricyanide/ferrocyanide ([Fe(CN)_6_]^3−^/[Fe(CN)_6_]^4−^) in 0.1 mol L^−1^ KCl (pH set at 7.4). As a proof of concept, the rGraphene-LIG electrode was applied for antibody determination in real samples using cyclic voltammetry, and a limit of detection (LOD) of 0.032 μg L^−1^ was achieved. When determining antigens in commercial samples, we obtained an LOD of 560 ηg mL^−1^ and a limit of quantification of 1677 ηg mL^−1^. The results of the electrochemical experiments were in accordance with the surface roughness obtained from atomic force microscopy images. Based on these results, the rGraphene-LIG electrode is shown to be an excellent platform for immunoglobulin detection when present in individuals after antigenic exposure caused by SARS-CoV-2.

## 1. Introduction

The COVID-19 pandemic, triggered by SARS-CoV-2, has led to the rapid development of accurate and efficient diagnostic tools. Among the various options, electrochemical biosensors stand out for their portability, low cost, and speed [1,2,3].

Severe acute respiratory syndrome, caused by a pathogen from the beta-coronavirus genus (SARS-CoV-2), has created a great challenge for humanity. Shortly after reports on the first cases of pneumonia of an unknown origin, which occurred in China, the world began to deal with the first major pandemic of the XXI century [4]. In this sense, scientific efforts were directed towards diverse areas of health so that the side effects of this disease were reversed, and there were investments in protein recognition, the mechanisms of action of the virus in the body, the production of antigens (Ag) and antibodies (Ac) on a large scale, prototypes for medicines, vaccines, diagnostics, and treatments [1,2,3].

In viral infections, type M immunoglobulins (IgM) are commonly present in the early stages of infections, while type G immunoglobulins (IgG) are recognized during later emergence and exhibit a prevalence in immunological memory processes. Thus, in COVID-19, immunoglobulins can be important biomarkers when analyzing the responses offered by the patient’s body to the infection [5]. Thus, since the beginning of the pandemic caused by SARS-CoV-2, several immunological and parasitological diagnoses have been developed, and we can observe the availability of gold-standard assays such as the enzyme-linked immunosorbent assay (ELISA) [6,7,8,9], C-reactive protein (PCR) [10], and rapid antigen tests (including self-tests) [11,12,13,14,15].

Electrochemical biosensors have advantages over other analysis systems, presenting diagnostic speed, portability, appropriate sensibility and selectivity, and a good cost–benefit ratio [16], and they can be applied even in regions with health systems with minimal infrastructure. In these devices, taking into consideration their wide range of properties, structures, and abundances, carbon-based materials have been used as a platform in an attempt to establish electrochemical diagnostics [17,18,19,20] as well as electrochemical biosensors for COVID-19 [21,22].

Since the emergence of the COVID-19 pandemic, several research groups have been developing diagnostic genosensors that establish the necessary reliability and portability [23,24]. In this sense, it is possible to observe works in which the polymers that helped in the detection of patients were printed onto the working electrodes; those describing the development of multiplex genosensors adapted to read the N and S proteins of SARS-CoV-2, presenting similar performance to the RT-qPCR technique [23]; and works describing the development of genosensors that utilize samples normally used in traditional tests, such as nasopharyngeal and salivary samples. In general, the diagnoses achieved by these groups are based on the viral genetic detection of patients affected by COVID-19 [16,22,24,25].

Laser-induced graphene electrodes (LIGs) on polyimide sheets provide interesting characteristics to improve diagnostic tests due to their unique properties, such as a three-dimensional macroporous structure, good conductivity and flexibility, a superior and facile laser fabrication process, and a low manufacturing cost [26,27,28]. In the construction process of these devices, sp^3^ carbons (from polyimide) are converted into sp^2^ carbons via a photothermal reaction, improving the electrical conductivity of the material and consequently the electrochemical performance. Thus, LIGs have been employed as substrates and modified with enzymes and aptamers, among other biological materials, to construct efficient biosensors [29,30].

The detection of anti-SARS-CoV-2 antibodies is essential in monitoring the evolution of the disease, evaluating the efficacy of vaccines, and monitoring the emergence of new variants. The biosensor proposed in this study represents a significant advancement in this area, offering a powerful tool in the management of the pandemic and in facing future threats to public health.

In this work, we produced a more cost-effective LIG platform for the detection of immunoglobulins in SARS-CoV-2. The LIG electrodes were subjected to different methodologies to adapt them to the profile of the biomolecule used, involving the electrochemical reduction of graphene. For comparison with the proposed diagnostic model, the enzyme-linked immunosorbent assay (ELISA), one of the techniques considered the gold standard for immunological detection, was used. Furthermore, to confirm the results and improve the readability of the diagnosis, artificial intelligence (AI) was incorporated into the development of this electrochemical biosensor.

## 2. Materials and Methods

### 2.1. Reagents and Instrumentation

All solutions were prepared using ultrapure water with resistivity greater than 18.2 MΩ cm (Millipore Corporation, Burlington, MA, USA). The used reagents were of analytical grade and without further purification (except commercial antigens). The biomolecule used as an antigen was the SARS-CoV-2 Spike Protein (S-RBD//a319–541) mFc Tag Recombinant Protein (Invitrogen, ThermoFisher Scientific, Waltham, MA, USA). For the electrochemical characterization of the immunosensor, 5.0 mmol L^−1^ [Fe(CN)_6_]^3−/4−^ and 0.1 mol L^−1^ KCl were used as a probe redox and supporting electrolyte, respectively.

The samples used in the experiments from patients who were positive for SARS-CoV-2 were authorized by the National Commission of Ethics in Research (CONEP) by Plataforma Brasil, under opinion number 3.957.676. All electrochemical measurements were carried out via cyclic voltammetry (CV) using EmStat 1 (PalmSens BV, The Netherlands) connected to a microcomputer at room temperature (25 ± 1 °C).

### 2.2. Fabrication of LIG Electrodes and Immunosensor

The LIG electrodes were produced on a polyimide sheet (ϕ 0.125 mm), obtained from Vemar (Sorocaba, Brazil), by laser engraving, as reported previously [26], using a blue laser with the power adjusted to 0.84 W and at a speed of 20 mm s^−1^. The proposed design consisted of a three-electrode configuration (working, counter, and pseudo-reference electrodes), similar to commercial screen-printed electrodes [31].

In these electrodes, we used a KCl solution, which acts as an ideal electrolyte, facilitating charge transfer between the electrode surface and the electrolyte solution and promoting the activation and reduction of stimuli coming from these electrodes. In this way, the presence of K^+^ ions in the solution reduces the interfacial resistance between the electrode and the electrolyte, optimizing the electron transfer and improving the electrochemical response. Activation with KCl increases the sensitivity and selectivity of LIG electrodes, allowing the precise detection of specific analytes with greater precision.

During the immunosensor development phase, we used some protocol variations, and the following immunosensor modifications and/or assemblies were tested.

(1) The LIG electrodes were reduced (rGraphene-LIG) and antigen immobilization was performed on the working electrode, followed by blocking with non-human protein and subsequent incubation with serum from an individual positive for COVID-19, containing anti-SARS-CoV-2 antibodies.
(2) The LIG electrodes were reduced (rGraphene-LIG) and the immobilization of the serum from the COVID-19-positive individual, containing anti-SARS-CoV-2 antibodies, was performed on the working electrode, followed by blocking with non-human protein and subsequent incubation with a commercial antigen, the SARS-CoV-2 Spike Protein (S-RBD//a319-541) mFc Tag Recombinant Protein (Invitrogen, ThermoFisher Scientific, Waltham, MA, USA).
(3) The LIG electrodes were modified with graphene oxide (GO) and then reduced (Graphene-rGO-LIG), and antigen immobilization was performed on the working electrode, followed by blocking with non-human protein and subsequent incubation with serum from a COVID-19-positive individual, containing anti-SARS-CoV-2 antibodies.
(4) The LIG electrodes were reduced, modified with graphene oxide, and then reduced again (rGraphene-rGO-LIG), and antigen immobilization was performed on the working electrode, followed by blocking with non-human protein and subsequent incubation with serum from individuals positive for COVID-19, containing anti-SARS-CoV-2 antibodies.

The reduction of graphene in the LIG electrodes into reduced graphene (called as r-Graphene-LIG in the text) was performed via cyclic voltammetric experiments in 0.1 mol L^−1^ KCl in the potential range of +0.5 V and −1.5 V (vs. Ag/AgCl), with a scan rate of 50 mV s^−1^ and step of 0.015 V. This procedure was adapted from the literature [32].

The immunosensor was built considering the antigen–antibody interaction. The immobilization of the biomolecules was carried out through adsorption; in this way, as a biomolecule was inserted, the conducting sites of the working electrode were blocked and, with this, a drop in current occurred. Thus, lower current readings were due to the immobilization of the initial biomolecule, and, when there was a detection, the current dropped even further. To avoid unspecific binding, a blocking solution (BS), was inserted after the adsorption of the initial biomolecule and washes were performed after all steps.

Thus, 80 µL of the redox probe solution, 5.0 mmol L^−1^ [Fe(CN)_6_]^3−/4^, was poured over the three electrodes, closing the circuit of the working electrode between the other two electrodes (counter electrode and reference), and the binding between the biomolecules was observed.

The percentages of oxidation and the reduction current were calculated from the initial CV (without the biomolecule), being considered as 100%.

### 2.3. Scanning Electron Microscopy (SEM)

The characterization of the morphology and composition of the laser-induced graphene electrodes (LIGs) used in the electrochemical biosensors was carried out using scanning electron microscopy (SEM) on a TESCAN VEJA 3LMU instrument. The LIG electrodes were fixed to aluminum stubs with conductive carbon tape to ensure good electrical contact during the analysis. For samples with low conductivity, metallization was performed with a thin layer of gold by sputtering.

The images were obtained under different operating conditions, adjusting the acceleration voltage, working distance, and beam current according to the characteristics of each sample. The magnification was adjusted to visualize the LIGs, from the general morphology to nanometric details. To identify the elements present in the LIGs and map their spatial distributions, energy-dispersive X-ray spectroscopy (EDS) was used.

### 2.4. Specificity

In addition to the tests performed on samples from individuals containing anti-protein S antibodies to SARS-CoV-2, pre-pandemic serum samples were used to check for possible nonspecific interactions.

### 2.5. Analytical Parameters

To evaluate the immunosensor’s sensitivity, an analytical curve with different dilutions of the serum from an individual positive for COVID-19 infection, containing anti-SARS-CoV-2 antibodies, was performed, namely pure serum, 1:300, 1:500, 1:1000, 1:1500, and 1:5000.

To check whether the electrochemical device’s detection depended on the SARS-CoV-2 antigen, a comprehensive analytical curve was obtained with various dilutions of the RBD SARS-CoV-2 antigen, namely 1 µg mL^−1^ and dilutions 40 ηg mL^−1^, 4 ηg mL^−1^, 2 ηg mL^−1^, and 0.2 ηg mL^−1^. This methodology aimed to guarantee the robustness and reliability of the results.

### 2.6. Interpreting Data by Artificial Intelligence (AI)

The pattern recognition in this work was developed through the Perceptron system. The data were obtained through the selection of voltammogram analysis; data mining was performed from the examination of information and the construction of existing relationships between the voltammograms generated by the electrochemical methodology and was executed using the deep learning system. The indices of interest were then consolidated to formulate the appropriate structure for the exploration and storage of the information. In this work, as algorithms, we used the Adam optimizer combined with the sigmoid function applied to the stochastic objective functions, and we applied values between 0 and 1 to define positive and negative patterns [33]. To increase the accuracy of the diagnostic analysis, the ReLu function was applied.

### 2.7. Indirect Enzyme-Linked Immunosorbent Assay (ELISA)

The standardization of the indirect ELISA for the determination of immunoglobulin G (IgG) anti-protein S of SARS-CoV-2 in serum samples from patients positive for the infection was also conducted.

High-affinity plates (Thermo Scientific^TM^ Nunc^TM^, EUA) were sensitized with the commercial antigen, SARS-CoV-2 Spike Protein (S-RBD/a319-541) and mFc Tag Recombinant Protein (Invitrogen, ThermoFisher Scientific, Waltham, Massachusetts, EUA) (0.2 μg mL^−1^), diluted in carbonate–bicarbonate buffer 0.06 mol L^−1^ (pH 9.5) and incubated for 18 h in 4 °C. After incubation, the plate was washed 3 times with a washing buffer PBS 1x (sodium phosphate saline solution with pH 7.4) containing 0.05% in Tween 20 (PBS-T) and blocked with PBS-T containing 5% skim powdered milk (Molico, Nestlé, São Paulo, SP, Brazil, PBS-T-M5 %) for 4 h at room temperature.

The plate was washed again and then the serum samples (1:100 diluted) were pipetted and incubated for 3 h at room temperature. After further washing, the anti-human IgG antibody (1:2000) conjugated with peroxidase (IgG/HRP, DAKO) was added and incubated for 2 h at room temperature. The conjugate was removed after further washing and the reaction was then revealed by the addition of the substrate 3,3′,5,5′-tetramethylbenzidine (ThermoFisher Scientific, Waltham, MA, USA).

Optical density (OD) values were determined in the microtiter plate reader at 450 nm. Antibody levels were expressed using the index ELISA (EI), according to the formula EI = DO sample/cut-off. The cut-off calculation was performed with the mean OD of the negative control serum (pre-pandemic serum) plus 3 standard deviations. EI values greater than or equal to 1.2 were considered positive, while borderline values close to EI = 1.0 were disregarded.

### 2.8. Statistical Analysis

To demonstrate the analyses, a comparative study of the voltammograms together with column graphs containing the peak current data, extracted from the CV measurements, was used.

## 3. Results and Discussion

**Figure 1:**
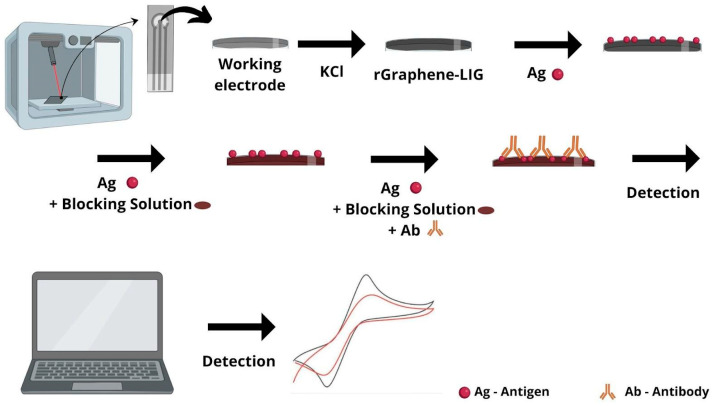
Schematic of rGraphene-LIG immunosensor. The manufacture of LIG electrodes was carried out using a device adapted to the Hypercube 3D printer. The steps for the preparation of the immunosensor were as follows. Electrochemical reduction in a KCl solution was conducted to improve the performance of the LIG electrodes. The rGraphene-LIG electrode was selected as the base platform; the SARS-CoV-2 antigen (Ag) was immobilized on the working electrode; the blocking solution was coupled to the platform; the antibodies (Ab—sample/real serum from the individuals) were coupled; and, at the end, the electroanalytical solution was inserted and the transduction process began.

The experiments carried out aimed at the characterization and development of an electrochemical immunosensor on an LIG platform for the detection of immunoglobulins produced against the SARS-CoV-2 virus during the acute phase of infection and/or monitoring the levels of antibodies after infection and/or vaccination. Figure 1 shows the preparation steps for a standardized immunosensor for the detection of anti-SARS-CoV-2 antibodies.

### 3.1. Reduced Graphene-LIG Electrodes

Our immunosensor used the receptor-binding domain (RBD) because it is responsible for binding the spike protein of SARS-CoV-2 to the angiotensin-converting enzyme 2 of human cells (hACE2) [34]. Thus, the experiments consisted of the standardization of the rGraphene-LIG immunosensor in order to guarantee the good detection of anti-SARS-CoV-2 antibodies in the sera of the individuals.

**Figure 2:**
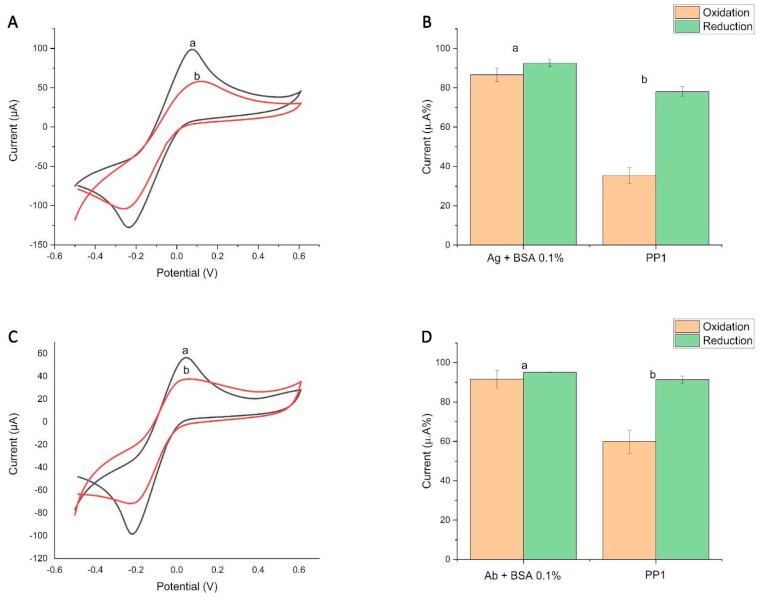
Graphic representing the initial value of the rGraphene-LIG immunosensor and the comparative methodology between (1) and (2) rGraphene-LIG diagnosis. (**A**,**B**) Cyclic voltammogram and column graph from (1) rGraphene-LIG indirect immunosensor extracted from cyclic voltammetry (CV) show the change in the current peak percentages (a) after the immobilization of the SARS-CoV-2 antigen (spike protein) followed by BS; (b) after the addition of sera from individuals with a known recent infection. (**C**,**D**) Cyclic voltammogram and column graph from (2) rGraphene-LIG immunosensor extracted from cyclic voltammetry (CV) show the change in the current peak percentages (a) after antibody immobilization (contained in the serum of an individual with a known recent infection) followed by BS; (b) after the addition of the SARS-CoV-2 antigen (spike protein). The percentages were calculated from the initial CV (without biomolecules), counted as 100%. The data denote oxidation in orange and reduction in green. The electrochemical signals of [Fe(CN)_6_]^4−^/[Fe(CN)_6_]^3−^ were obtained at a scan rate of 100 mV s^−1^. PP1 = Positive Patient 1.

Initial tests with (1) rGraphene-LIG were performed on working electrodes treated with 0.1 mol L^−1^ KCl and demonstrated the good detection of antibodies in the serum of an individual known to be positive for this infection (Figure 2A,B). These results corroborate research that points to the efficiency of graphene electrodes when compared to other carbonaceous materials, since graphene increases the surface area and offers the ability to bind molecules through physical adsorption on the electrodes used in the construction of electrochemical biosensors [19].

To check whether we could also build a device using the patient’s serum containing anti-SARS-CoV-2 antibodies as a probe to search for SARS-CoV-2 antigens (analyte), we tested the possibility of initially inserting the individual’s serum into the (2) rGraphene-LIG electrode, leaving the commercial antigen for the detection step. Since patients’ serum represents a readily available source of biological probes, it eliminates the need for the purification or modification of commercial types of probes.

In this experiment, we verified that there was detection, but the inferiority of the diagnostic parameters was observed when compared to the indirect immunosensor (Figure 2C,D). It is possible that this occurred because patients’ serum is a complex, protein-rich compound. In percentage proportions, albumins represent around 60% of the protein components of this sample, while globulins comprise the other 40%, and, among the globulins, we can observe the presence of immunoglobulins G (IgGs) that represent up to 20% of this compound [35]. Therefore, improvements related to direct immunosensing require the greater purification and pre-treatment of samples, which may hinder the global application of these diagnoses in remote and resource-poor areas.

### 3.2. Graphene-GO-LIG Electrodes

**Figure 3:**
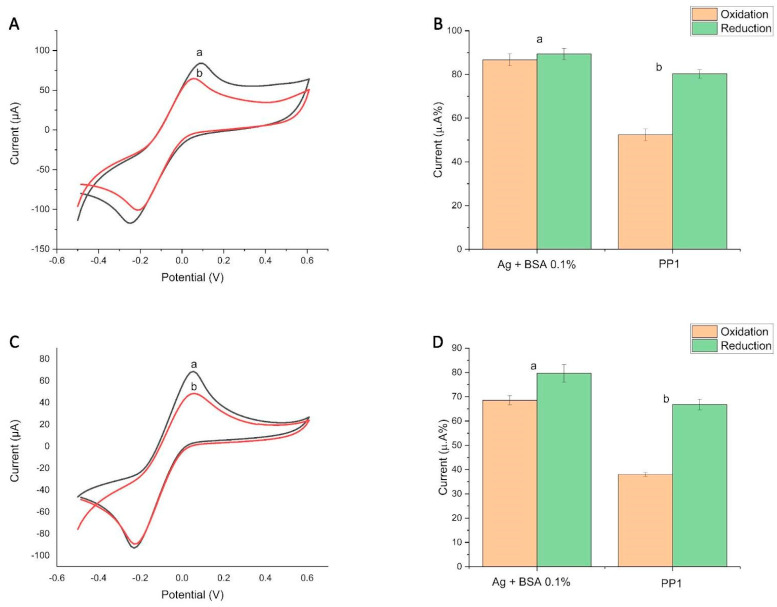
Comparison of Graphene-GO-LIG modification with (3) graphene oxide (GO) without prior reduction and with (4) reduction before and after GO adsorption. (**A**,**B**) Cyclic voltammogram and column graph extracted from cyclic voltammetry (CV) show the change in the current peak percentages (a) after the immobilization of the SARS-CoV-2 antigen (spike protein) followed by BS and (b) after the addition of sera from individuals with a known recent infection in (3) Graphene-rGO-LIG. (**C**,**D**) Cyclic voltammogram and column graph extracted from cyclic voltammetry (CV) show the change in the current peak percentages (a) after the immobilization of the SARS-CoV-2 antigen (spike protein) followed by BS and (b) after the addition of sera from individuals with a known recent infection in (4) rGraphene-rGO-LIG. The percentages were calculated from the initial CV (without biomolecules), counted as 100%. The data denote oxidation in orange and reduction in green. The electrochemical signals of [Fe(CN)_6_]^4−^/[Fe(CN)_6_]^3−^ were obtained at a scan rate of 100 mV s^−1^. PP1 = Positive Patient 1.

Another possibility for improvement was the application of 0.5 mg mL^−1^ GO solution, due to its ability to increase the surface area of the electrode. For such tests, we evaluated the effectiveness of the modification with the adsorption of GO directly on the electrode followed by reduction—(3) Graphene-rGO-LIG(Figure 3A,B)—and also by performing reduction before and after GO adsorption—(4) rGraphene-rGO-LIG (Figure 3C,D). When comparing the results, we observed a slight improvement after performing the reduction before and after graphene adsorption ((4) rGraphene-rGO-LIG immunosensor).

However, the modification with GO, in both (3) and (4), did not improve the immunosensor, because, when we compared the results for (1) rGraphene-LIG (Figure 2A,B), which was the best platform among the Graphene-LIGs, and (4) rGraphene-rGO-LIG (Figure 3C,D), which was the best platform among the Graphene-GO-LIGs, we could observe that the best performance in the tests occurred for (1) rGraphene-LIG (Figure 2A,B). Reduced graphene oxide (rGO) has good conductive characteristics, but, in aqueous solutions, the aggregations generated by cohesive interactions make it difficult to disperse this material, especially in polymer matrices, where the interfacial interactions become fragile [36,37]. After the analysis, we chose to continue with the electrochemical device without the GO modification, since we did not observe significant statistical differences between the two platforms (Figure 3A–D). This decision was made based on several factors that considered the feasibility and social impact of our test in detecting SARS-CoV-2.

### 3.3. Specificity and Comparison with Indirect ELISA

**Figure 4:**
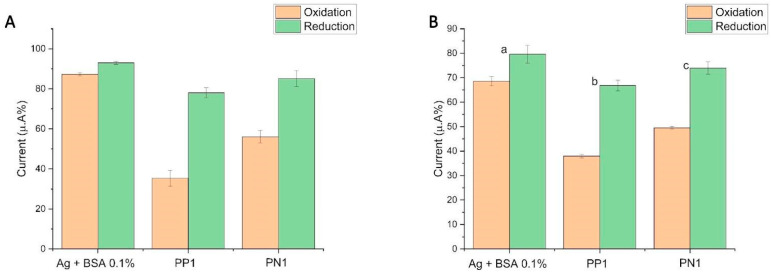
Comparison of (**A**) rGraphene-LIG electrode and (**B**) rGO-LIG, respectively. Column graphs extracted from cyclic voltammetry (CV) analyses show the change in the current peak percentages (a) after the immobilization of the SARS-CoV-2 antigen (spike protein) followed by BS; (b) after the addition of sera from individuals with a known recent infection; (c) after the addition of pre-pandemic serum (negative for SARS-CoV-2). The percentages were calculated from the initial CV (without biomolecules), counted as 100%. The data denote oxidation in orange and reduction in green. The electrochemical signals of [Fe(CN)_6_]^4−^/[Fe(CN)_6_]^3−^ were obtained at a scan rate of 100 mV s^−1^.

Seeking to challenge the devices developed, we chose to analyze their performance by preparing a pre-pandemic serum, which was negative for SARS-CoV-2 infection; we observed an interaction in all models of the platform (Figure 4A,B). In immunology, we refer to antigens as the constituent proteins of a microorganism that are capable of triggering an immune system response.

Each antigen can have several epitopes, which are sequences of amino acids that are recognized by cells or antibodies (also called immunoglobulins) in the adaptive immune response, so that there is a specific response for the type of microorganism causing the infection [38,39]. Although antibodies have specificity, in some cases, nonspecific binding may occur due to the fact that the same antibody recognizes two completely different epitopes that are structurally similar (molecular mimicry) [40,41,42]. This nonspecific recognition can occur both between epitopes of different microorganisms and when an antibody specific to a pathogen mistakenly binds to the individual’s own antigens.

In terms of SARS-CoV-2 infection, this could be a plausible explanation for the nonspecific interaction presented in our immunosensor. A study by Shiakolas and colleagues (2021) analyzed a sample of peripheral blood mononuclear cells (PBMC) from an individual affected by SARS-CoV ten years before the collection was performed and observed that cross-reactivity occurred between other coronaviruses and SARS-CoV-2 in several protein S epitopes, including the receptor-binding domain (RBD) that was tested in our study, although this was a weak interaction [43].

Tests on serum samples from patients who had contracted SARS-CoV years before, performed by ELISA, also demonstrated strong cross-reactivity with SARS-CoV-2 for both the nucleocapsid protein (N); the S, S2, and S1 proteins; and the RBD of the spike protein, although the reactivity between the two was lower [44].

**Figure 5:**
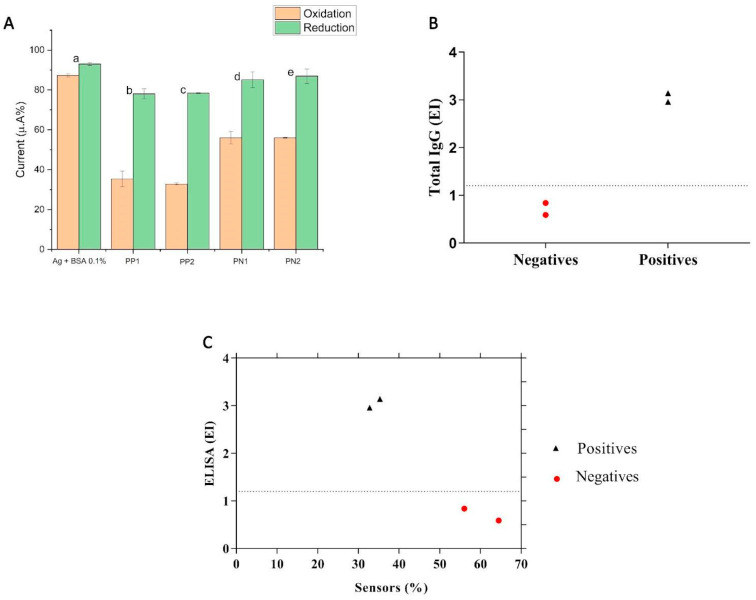
Comparison between rGraphene-LIG immunosensor and ELISA results. (**A**) The column graph extracted from cyclic voltammetry (CV) shows the change in the current peak percentages (a) after the immobilization of the SARS-CoV-2 antigen (spike protein) followed by BS; (b) and (c) after the addition of serum from an individual with a known recent infection; (d) and (e) after the addition of pre-pandemic serum. The data denote oxidation in orange and reduction in green. (**B**) Indirect ELISA. (**C**) Correlation between levels of total IgG antibodies in ELISA and percentage of currents (mA) in electrochemical tests. The horizontal line represents the point where individuals are considered positive in the ELISA, with EI = 1.2. PP1 = Positive Patient 1; PP2 = Positive Patient 2; PN1 = Negative Patient 1; PN2 = Negative Patient 2.

ELISA is commonly used to validate immunosensors, as it is a well-established technique in serological diagnoses. Thus, the results obtained with the developed biosensor (Figure 5A) were also confirmed by this technique (Figure 5B). Both techniques were in agreement (Figure 5C). However, in our experiments, we observed that the electrochemical immunosensor was able to demonstrate the presence of mild nonspecific interactions not identified in the ELISA; this can be explained by the good sensitivity of the biosensor when compared to other available techniques for the serological detection of patients and the initial “n” for the ELISA that was considered low for this analytical technique.

Patients who had recovered from the disease caused by SARS-CoV also had T cells with high cross-reactivity with the N protein from SARS-CoV-2 [41]. Interestingly, individuals not exposed to SARS or COVID-19 also showed this cross-reactivity [45]. Previous studies have demonstrated pre-existing CD4+ T cell reactivity to SARS-CoV-2 in a substantial proportion of unexposed individuals, with Grifoni et al. (2020) reporting recognition rates of 40–60% and Braun et al. observing similar responses in approximately 35% of their study [46,47].

Sequence homology between SARS-CoV-2 and other human coronaviruses (HCoVs) has been observed and this sequence similarity likely contributes to the presence of pre-existing memory CD4+ T cells that can recognize SARS-CoV-2 epitopes in individuals with no known prior exposure to the virus [48], which are circulating in several countries. In the United States, for example, between 2014 and 2017, more than 850,000 tests were performed for HCoV, reported in 117 laboratories in 42 states, of which almost 30,000 were positive [49]. Thus, considering the number of circulating coronaviruses and the presence of other reports of the previous detection of cells and antibodies showing cross-reactivity with SARS-CoV-2, this may be one of the reasons for the presence of weak antigen–antibody interactions detected in the negative sera in our tests.

Another explanation for the nonspecific interactions demonstrated in the sera of negative individuals would be the presence of the soluble human protein angiotensin-converting enzyme 2 (sACE2) binding to the RBD-S antigen used. It has already been shown that the RBD strongly binds to soluble ACE2 from both humans and bats and that soluble ACE2 could block the binding between the SARS-CoV-2 RBD and its cellular receptor, hACE2 [50], acting as a neutralizing agent regarding virus invasion into cells in vitro [51,52]. Thus, it is possible that soluble ACE2 may be present in the sera of negative individuals, binding to the RBD present in the immunosensor; however, more tests and a greater number of samples are needed to test this hypothesis.

### 3.4. Analytical Curve

**Figure 6:**
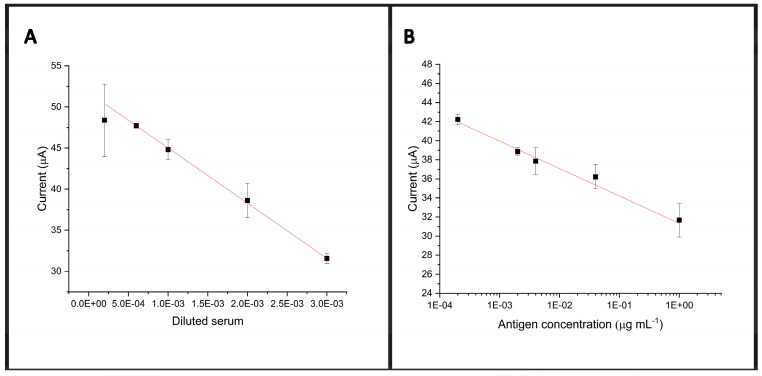
Analytical curves obtained from the percentages of the current peaks for triplicate measurements of the biosensors in the presence of serum from an individual positive for coronavirus infection (**A**) containing anti-SARS-CoV-2 antibodies, in pure serum, and in dilutions of 1:300, 1:500, 1:1000, 1:1500, and 1:5000. Analytical curves obtained from the percentages of the current peaks for triplicate measurements of the biosensors in the presence of RBD antigen (**B**) in 1 µg mL^−1^ and in dilutions of 40 ηg mL^−1^, 4 ηg mL^−1^, 2 ηg mL^−1^, and 0.2 ηg mL^−1^ (**B**). PP1 = Positive Patient 1.

Figure 6 demonstrates the preliminary results of the analytical curve using the immunosensor. In Figure 6A, the current of the redox probe is inversely proportional to the concentration of antibodies. We used the following serum dilutions from individuals positive for coronavirus infection: 1:300, 1:500, 1:1000, 1:1500, and 1:5000 to perform the antibody calibration curve. Thus, our results presented a correlation coefficient of 0.9994 (for the equation: i(%) = −727.5 × [serum dilution ratio] + 72.83). The equation was obtained from the linear regression of the peak current (%) vs. the RBD-SARS-CoV-2 concentration. As a proof of concept, the rGraphene-LIG electrode was applied for antibody determination in real samples using cyclic voltammetry with a limit of detection (LOD) of 0.032 μg L^−1^. In Figure 6B, the redox probe current is inversely proportional to the concentration of antigens. We used serial dilutions of the SARS-CoV-2 Spike Protein (S-RBD/a319-541) and mFc Tag Recombinant Protein at 1 µg mL^−1^ and dilutions of 40 ηg mL^−1^, 4 ηg mL^−1^, 2 ηg mL^−1^, and 0.2 ηg mL^−1^ (tests carried out in triplicate). This graph presents a correlation coefficient of 0.9798, using an equation obtained from the linear regression of the current peak (%) vs. the concentration of anti-SARS-CoV-2 antibodies. As a proof of concept, the Graphene-LIG electrode was applied to determine antigens in commercial samples using cyclic voltammetry, with an LOD of 560 ηg mL^−1^ and a limit of quantification of 1677 ηg mL^−1^. Supplementary Table S1 compares the obtained results in this work with those for some other biosensors for SARS-CoV-2 detection and their characteristics.

### 3.5. Surface Characterization by Atomic Force Microscopy (AFM)

**Figure 7:**
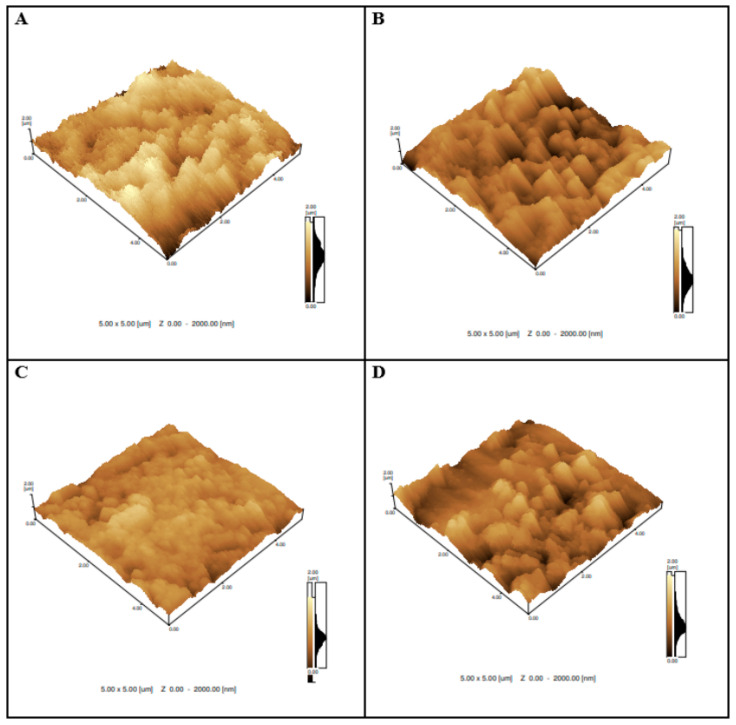
AFM images of (**A**) graphene; (**B**) graphene/RBD + BSA; (**C**) graphene/RBD + BSA/positive patient; and (**D**) graphene/RBD + BSA/negative patient. Roughness values obtained by AFM were 309 nm (bare graphene electrode), 235 nm (graphene/RBD + BSA), 166 nm (graphene/RBD + BSA/positive patient), and 220 nm (graphene/RBD + BSA/negative patient).

As shown in Figure 7B, the immobilization of the RBD produced a decrease in the height and size of the clusters when compared to the electrode without biomolecules (Figure 7A), indicating that the RBD was successfully incorporated onto the electrode surface. The electrodes containing graphene RBD + BSA/positive patient (Figure 7C) showed topographies that were more homogenous, suggesting the occurrence of the hybridization event, in agreement with the results obtained in the electrochemical studies. Meanwhile, graphite/RBD + BSA/negative patient (Figure 7D) showed topographies with a globular aspect, but the latter was less homogenous, presenting larger clusters as in Figure 7B, suggesting the absence of the hybridization event.

Thus, for the comparison and monitoring of potentially neutralizing anti-RBD antibodies after SARS-CoV-2 infection, the development and characterization of this electrochemical biosensor performed on rGraphene-LIG electrodes proved it to be efficient.

In our results, we analyzed the correlation between the morphology of the LIG and the electrochemical performance obtained in the proposed biosensor. SEM proved to be an essential tool for the characterization of the LIGs, providing detailed information about their morphologies and compositions. The clarity between the structural characteristics of the LIGs and the performance of the biosensors allowed a better understanding of the working mechanisms of these devices and contributed to the development of new biosensors with greater sensitivity and selectivity.

**Figure 8:**
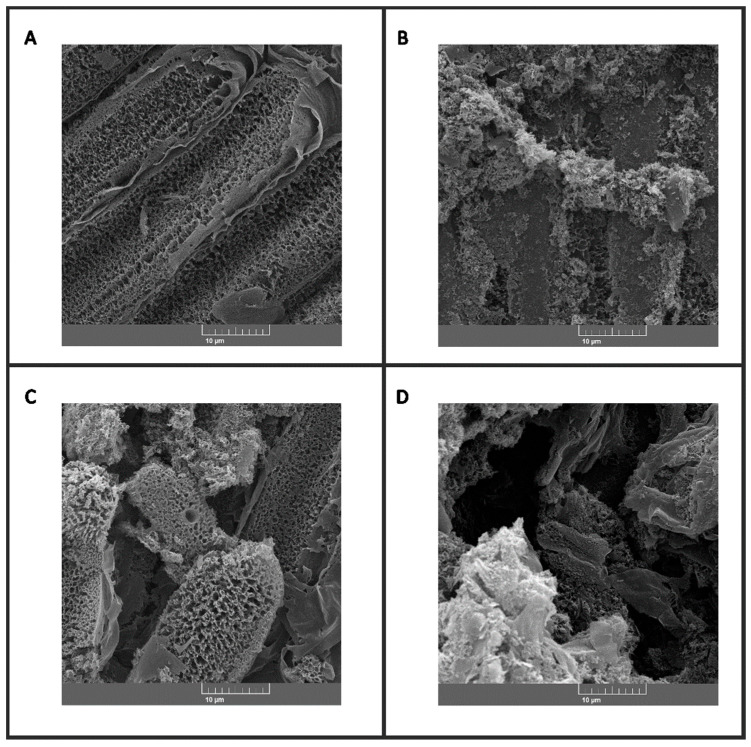
Scanning electron micrographs (SEM) of graphene electrodes modified with different biomolecules in (**A**) graphene; (**B**) graphene/RBD + BSA; (**C**) graphene/RBD + BSA/positive patient; and (**D**) graphene/RBD + BSA/negative patient. The images reveal the surface morphologies of the electrodes, highlighting the presence of porous and rough structures characteristic of graphene, as well as the interaction of the biomolecules with the electrode surface. Scale: 10 µm.

The SEM micrographs presented in Figure 8A–D demonstrate the characteristic morphologies of the laser-induced graphene electrodes (LIGs) used in this study. The porous and rough structure observed is the result of the laserization process, which promotes the formation of a network of interconnected pores, increasing the surface area of the material and consequently its adsorption and charge transfer capacity [52,53]. This morphology is fundamental to the performance of LIGs as electrodes in electrochemical devices, such as the biosensor developed in this work.

The comparison between the SEM images and the electrochemical results obtained allowed the establishment of a correlation between the morphologies of the LIGs and the performance of the sensors. The large surface areas and porous structures of the LIGs contributed to the greater sensitivity and selectivity of the biosensor, facilitating the detection of biomarkers of interest.

Scanning electron micrographs (SEM) of graphene electrodes modified with different biomolecules were obtained for (A) graphene; (B) graphene/RBD + BSA; (C) graphene/RBD + BSA/positive patient; and (D) graphene/RBD + BSA/negative patient. The images reveal the surface morphologies of the electrodes, highlighting the presence of porous and rough structures characteristic of graphene, as well as the interaction of the biomolecules with the electrode surface (scale: 10 µm). Figure 8A shows the parallel conductive tracks engraved by the laser, typically obtained for LIG electrodes. Figure 8B–D show the surfaces covered by the respective modifiers added to the electrode surface. The conductive tracks shown in Figure 8A were completely covered after the biosensor’s construction.

### 3.6. Artificial Intelligence

In studies involving clinical diagnoses, sometimes, it is necessary for qualified professionals to monitor specific parameters of the device, which brings difficulty in commercializing these diagnoses at a low operational cost. From this point of view, the development of software that simplifies the analysis of the results and maintains quality in the recognition of positive and negative patients is a contemporary demand [54,55].

In this sense, envisioning the development of diagnostic tests that are cost-effective and commercially possible for interfaces created using biosensors, an initial model of a neural network (Figure 8) has been proposed that makes it possible to use a commercial device for the diagnosis or monitoring of specific anti-SARS-CoV-2 immunoglobulins. In the input layer of this artificial intelligence model, 10 coupled neurons were used to analyze the electroanalytical reading patterns and obtain the analyzed biomolecules. Therefore, the output layer was developed with one neuron with a sigmoid activation function programmed to evaluate the positivity or negativity of the patients presented.

**Figure 9:**
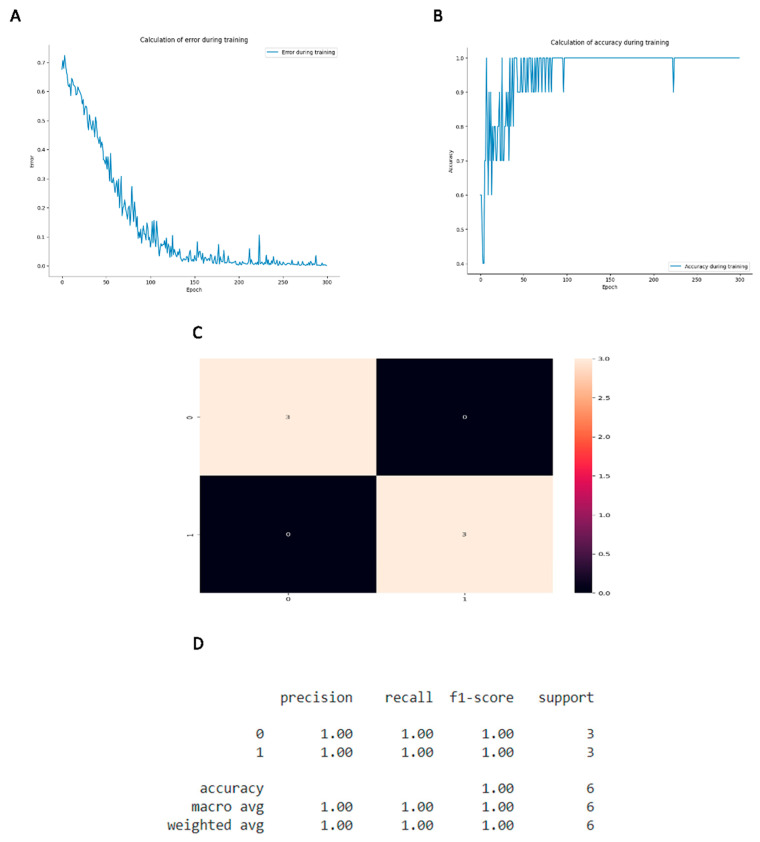
Development and application of artificial intelligence (AI) to improve diagnostic results. (**A**) Graph representing the likelihood of errors in the methodology applied for the diagnostic evaluation of patients. (**B**) Graph indicating the precision model presented for the applied diagnostic methodology. (**C**) Assessment matrix for the diagnosis of the evaluated disease. (**D**) Table representing the mean diagnostic accuracy presented by the developed artificial intelligence.

The rigor of the developed method was evaluated through error calculation (Figure 9A). Results with indices close to zero on the “y-axis”, as demonstrated in this work, are related to greater efficiency in dealing with the data presented. To assess the accuracy of the method developed, an analysis chart was generated (Figure 9B), so that results close to 1 on the “y-axis”, as presented in this work, indicate a higher rate of correct answers in the process of creating the training model.

The developed confusion matrix (Figure 9C) sought to present the results from the neural network in a more accessible way; thus, the methodology developed proved to be efficient for the number of patients presented to the interface. Here, when we presented patients with the presence of anti-SARS-CoV-2 immunoglobulins and pre-pandemic sera, this software was able to obtain correct results. Thus, these results indicated average precision of 100% (Figure 9D) for the proposed data. In this way, our results demonstrate the potential of the artificial intelligence model developed in the detection of positivity and negativity in patients.

## 4. Conclusions

The COVID-19 pandemic has highlighted the urgent need to strengthen the resilience of health systems to face new threats to public health. Investments in the research and development of innovative technologies are crucial. The results of this study demonstrate the potential of LIG electrode biosensors as a powerful tool for the rapid and accurate detection of neutralizing antibodies against SARS-CoV-2.

This study demonstrates the potential of an electrochemical immunosensor based on r-LIG electrodes for the detection of immunoglobulins produced against SARS-CoV-2. The sensor demonstrated high sensitivity, with detection limits of 0.032 μg∙L⁻¹ for antibodies and 560 ηg∙mL⁻¹ for antigens. The r-LIG platform outperformed conventional methods in terms of speed (cyclic voltammetry analysis) and simplicity (avoiding the need for fluorophore or enzyme labeling).

In addition, AFM and SEM characterization revealed visual evidence of the successful immobilization of the RBD antigen on the electrode surface. Furthermore, the introduction of artificial intelligence in the data analysis allowed the automation of the results’ interpretation, making the diagnosis faster and more accessible.

Although initial tests with patient serum as a probe source for SARS-CoV-2 antigen detection showed promising results, indirect immunodetection using a purified viral antigen proved to be more efficient and standardizable. Despite the nonspecific detection observed in some SARS-CoV-2-negative sera, possibly due to cross-reactivity with other coronaviruses, the sensor demonstrated high specificity for positive samples. Future research advancing the implementation of gating strategies to minimize cross-reactivity will be essential to improve the sensor.

Thus, this work presents a promising electrochemical immunosensor for the detection of anti-SARS-CoV-2 antibodies. The combination of high sensitivity, speed, simplicity, and integration with artificial intelligence makes this technology an attractive tool for the diagnosis of COVID-19 in different scenarios, including point-of-care and resource-limited settings.

## Data Availability

Data are contained within the article and Supplementary Materials.

## Supplementary Materials

The following supporting information can be downloaded at: https://www.mdpi.com/article/10.3390/bios14110514/s1, Table S1: Comparison of SARS-COV-2 detection methods.

## References

[1] Li G., Hilgenfeld R., Whitley R., De Clercq E. (2023). Therapeutic strategies for COVID-19: Progress and lessons learned. Nat. Rev. Drug Discov..

[2] Majumder J., Minko T. (2021). Recent Developments on Therapeutic and Diagnostic Approaches for COVID-19. AAPS J..

[3] Tregoning J.S., Flight K.E., Higham S.L., Wang Z., Pierce B.F. (2021). Progress of the COVID-19 vaccine effort: Viruses, vaccines and variants versus efficacy, effectiveness and escape. Nat. Rev. Immunol..

[4] Hafeez A., Ahmad S., Siddqui S.A., Ahmad M., Mishra S. (2020). A Review of COVID-19 (Coronavirus Disease-2019) Diagnosis, Treatments and Prevention. Eurasian J. Med. Oncol..

[5] Liu X., Wang J., Xu X., Liao G., Chen Y., Hu C.H. (2020). Patterns of IgG and IgM antibody response in COVID-19 patients. Emerg. Microbes Infect..

[6] Van Elslande J., Houben E., Depypere M., Brackenier A., Desmet S., Andr E., Ranst M.V., Lagrou K., Vermeersch P. (2020). Diagnostic performance of seven rapid IgG/IgM antibody tests and the Euroimmun IgA/IgG ELISA in COVID-19 patients. Clin. Microbiol. Infect..

[7] Gdoura M., Ben Ghaloum F., Hamida M.B., Chamsa W., Triki H., Bahloul C. (2022). Development of an in—House quantitative ELISA for the evaluation of different COVID-19 vaccines in humans. Sci. Rep..

[8] Luo J., Brakel A., Krizsan A., Ludwig T., Mötzing M., Volke D., Lakowa N., Grünewald T., Lehmann C., Wolf J. (2022). Sensitive and specific serological ELISA for the detection of SARS-CoV-2 infections. Virol. J..

[9] Sapkal G., Shete-aich A., Jain R., Yadav P.D., Sarkale P., Lakra R., Baradkar S., Deshpande G.R., Mali D., Tilekar B.N. (2020). Development of indigenous IgG ELISA for the detection of anti-SARS-CoV-2 IgG. Indian J. Med. Res..

[10] Tombuloglu H., Sabit H., Al-Suhaimi E., Al Jindan R., Alkharsah K.R. (2021). Development of multiplex real-time RT-PCR assay for the detection of SARS-CoV-2. PLoS ONE.

[11] Feng M., Chen J., Xun J., Dai R., Zhao W., Lu H., Xu J., Chen L. (2020). Development of a Sensitive Immunochromatographic Method Using Lanthanide Fluorescent Microsphere for Rapid Serodiagnosis of COVID-19. ACS Sens..

[12] Jian M., Perng C., Chung H., Chang C., Lin J., Yeh K.-M., Chen C.-W., Hsieh S.-S., Pan P.-C., Chang H.-T. (2022). Clinical assessment of SARS-CoV-2 antigen rapid detection compared with RT-PCR assay for emerging variants at a high-throughput community testing site in Taiwan. Int. J. Infect. Dis..

[13] Kashiwagi K., Ishii Y., Aoki K., Yagi S., Maeda T., Miyazaki T., Yoshizawa S., Aoyagi K., Tateda K. (2021). Immunochromatographic test for the detection of SARS-CoV-2 in saliva. J. Infect. Chemother..

[14] Pan Y., Li X., Yang G., Fan J., Tang Y., Zhao J. (2020). Serological immunochromatographic approach in diagnosis with SARS-CoV-2 infected COVID-19 patients. J. Infect..

[15] Tozetto-mendoza T.R., Kanunfre K.A., Vilas-boas L.S., Espinoza E.P.S., Paião H.G.O., Rocha M.C., de Paula A.V., de Oliveira M.S., Zampelli D.B., Vieira J.M. (2021). Nucleoprotein-based ELISA for detection of SARS-COV-2 IgG antibodies: Could an old assay be suitable for serodiagnosis of the new coronavirus?. J. Virol. Methods.

[16] Torres M.D.T., de Araujo W.R., de Lima L.F., Ferreira A.L., de la Fuente-Nunez C. (2021). Low-cost biosensor for rapid detection of SARS-CoV-2 at the point of care. Matter.

[17] Beduk T., Beduk D., De Oliveira I., Zihnioglu F., Cicek C., Sertoz R. (2021). Rapid Point-of-Care COVID-19 Diagnosis with a Gold-Nanoarchitecture-Assisted Laser-Scribed Graphene Biosensor. Anal. Chem..

[18] Martins B.R., Barbosa Y.O., Andrade C.M.R., Pereira L.Q., Simão G.F., de Oliveira C.J., Correia D., Oliveira R.T.S., da Silva M.V., Silva A.C.A. (2020). Development of an electrochemical immunosensor for specific detection of visceral leishmaniasis using gold-modified screen-printed carbon electrodes. Biosensors.

[19] Segundo J.E.D.V., Vilar E.O. (2016). Grafeno: Uma revisão sobre propriedades, mecanismos de produção e potenciais aplicações em sistemas energéticos. Rev. Eletrônica Mater. E Process..

[20] Tan A.Y.S., Lo N.W., Cheng F., Zhang M., Tan M.T.T., Manickam S., Muthoosamy K. (2023). 2D carbon materials based photoelectrochemical biosensors for detection of cancer antigens. Biosens. Bioelectron..

[21] Alafeef M., Dighe K., Moitra P., Pan D. (2020). Rapid, Ultrasensitive, and Quantitative Detection of SARS-CoV-2 Using Antisense Oligonucleotides Directed Electrochemical Biosensor Chip. ACS Nano.

[22] Ayankojo A.G., Boroznjak R., Reut J., Opik A., Syritski V. (2022). Molecularly imprinted polymer based electrochemical sensor for quantitative detection of SARS-CoV-2 spike protein. Sens. Actuators B Chem..

[23] Chaibun T., Puenpa J., Ngamdee T., Boonapatcharoen N., Athamanolap P., O’Mullane A.P., Vongpunsawad S., Poovorawan Y., Lee S.Y., Lertanantawong B. (2021). Rapid electrochemical detection of coronavirus SARS-CoV-2. Nat. Commun..

[24] Kashefi-Kheyrabadi L., Nguyen H.V., Go A., Baek C., Jang N., Lee J.M., Cho N.H., Min J., Lee M.H. (2022). Rapid, multiplexed, and nucleic acid amplification-free detection of SARS-CoV-2 RNA using an electrochemical biosensor. Biosens. Bioelectron..

[25] Daniels J., Wadekar S., DeCubellis K., Jackson G.W., Chiu A.S., Pagneux Q., Saada H., Engelmann I., Ogiez J., Loze-Warot D. (2021). A mask-based diagnostic platform for point-of-care screening of COVID-19. Biosens. Bioelectron..

[26] Costa W.R.P., Rocha R.G., de Faria L.V., Matias T.A., Ramos D.L.O., Dias A.G.C., Fernandes G.L., Richter E.M., Muñoz R.A.A. (2022). Affordable equipment to fabricate laser-induced graphene electrodes for portable electrochemical sensing. Microchim. Acta.

[27] Lin J., Peng Z., Liu Y., Ruiz-Zepeda F., Ye R., Samuel E.L.G., Yacaman M.J., Yakobson B.I., Tour J.M. (2014). Laser-induced porous graphene films from commercial polymers. Nat. Commun..

[28] Matias T.A., de Faria L.V., Rocha R.G., Silva M.N.T., Nossol E., Richter E.M., Muñoz R.A.A. (2022). Prussian blue-modified laser-induced graphene platforms for detection of hydrogen peroxide. Microchim. Acta.

[29] Dixit N., Singh S.P. (2022). Laser-Induced Graphene (LIG) as a Smart and Sustainable Material to Restrain Pandemics and Endemics: A Perspective. ACS Omega.

[30] Wan Z., Nguyen N.T., Gao Y., Li Q. (2020). Laser induced graphene for biosensors. Sustain. Mater. Technol..

[31] Nascimento V.B. (1998). Eletrodos Fabricados Por “Silk-Screen”. Quimica Nova.

[32] Rocha D.P., Dornellas R.M., Cardoso R.M., Narciso L.C.D., Silva M.N.T., Nossol E., Richter E.M., Munoz R.A.A. (2018). Chemically versus electrochemically reduced graphene oxide: Improved amperometric and voltammetric sensors of phenolic compounds on higher roughness surfaces. Sens. Actuators B Chem..

[33] Barakat A., Bianchi P. (2021). Convergence and Dynamical Behavior of the ADAM Algorithm for Nonconvex Stochastic Optimization. SIAM J. Optim..

[34] Shang J., Wan Y., Luo C., Ye G., Geng Q., Auerbach A., Li F. (2020). Cell entry mechanisms of SARS-CoV-2. Proc. Natl. Acad. Sci. USA.

[35] Leeman M., Choi J., Hansson S., Storm M.U., Nilsson L. (2018). Proteins and antibodies in serum, plasma, and whole blood—Size characterization using asymmetrical flow field-flow fractionation (AF4). Anal. Bioanal. Chem..

[36] Layek R.K., Nandi A.K. (2013). A review on synthesis and properties of polymer functionalized graphene. Polymer.

[37] Li D., Kaner R.B. (2008). Materials science: Graphene-based materials. Science.

[38] Davies D.R., Metzger H. (1983). Structural Basis of Antibody Function. Annu. Rev. Immunol..

[39] Sela-Culang I., Kunik V., Ofran Y. (2013). The structural basis of antibody-antigen recognition. Front. Immunol..

[40] Wrapp D., Wang N., Corbett K.S., Goldsmith J.A., Hsieh C.L., Abiona O., Graham B.S., McLellan J.S. (2020). Cryo-EM structure of the 2019-nCoV spike in the prefusion conformation. Science.

[41] Lu R., Zhao X., Li J., Niu P., Yang B., Wu H., Wang W., Song H., Huang B., Zhu N. (2020). Genomic characterisation and epidemiology of 2019 novel coronavirus: Implications for virus origins and receptor binding. Lancet.

[42] Gao T., Gao Y., Liu X., Nie Z., Sun H., Lin K., Peng H., Wang S. (2021). Identification and functional analysis of the SARS-COV-2 nucleocapsid protein. BMC Microbiol..

[43] Shiakolas A.R., Kramer K.J., Wrapp D., Richardson S.I., Schäfer A., Wall S., Wang N., Janowska K., Pilewski K.A., Venkat R. (2021). Cross-reactive coronavirus antibodies with diverse epitope specificities and Fc effector functions. Cell Rep. Med..

[44] Zhu Y., Yu D., Han Y., Yan H., Chong H., Ren L., Wang J., Li T., He Y. (2020). Cross-reactive neutralization of SARS-CoV-2 by serum antibodies from recovered SARS patients and immunized animals. Sci. Adv..

[45] Le Bert N., Tan A.T., Kunasegaran K., Tham C.Y.L., Hafezi M., Chia A., Chng M.H.Y., Lin M., Tan N., Linster M. (2020). SARS-CoV-2-specific T cell immunity in cases of COVID-19 and SARS, and uninfected controls. Nature.

[46] Braun J., Loyal L., Frentsch M., Wendisch D., Georg P., Kurth F., Hippenstiel S., Dingeldey M., Kruse B., Fauchere F. (2020). SARS-CoV-2-reactive T cells in healthy donors and patients with COVID-19. Nature.

[47] Grifoni A., Weiskopf D., Ramirez S.I., Mateus J., Dan J.M., Moderbacher C.R., Rawlings S.A., Sutherland A., Premkumar L., Jadi R.S. (2020). Targets of T Cell Responses to SARS-CoV-2 Coronavirus in Humans with COVID-19 Disease and Unexposed Individuals. Cell.

[48] Mateus J., Grifoni A., Tarke A., Sidney J., Ramirez S.I., Dan J.M., Burger Z.C., Rawlings S.A., Smith D.M., Phillips E. (2020). Selective and cross-reactive SARS-CoV-2 T cell epitopes in unexposed humans. Science.

[49] Killerby M.E., Biggs H.M., Haynes A., Dahl R.M., Mustaquim D., Gerber S.I., Watson J.T. (2018). Human coronavirus circulation in the United States 2014–2017. J. Clin. Virol..

[50] Tai W., He L., Zhang X., Pu J., Voronin D., Jiang S., Zhou Y., Du L. (2020). Characterization of the receptor-binding domain (RBD) of 2019 novel coronavirus: Implication for development of RBD protein as a viral attachment inhibitor and vaccine. Cell. Mol. Immunol..

[51] Monteil V., Kwon H., Prado P., Hagelkrüys A., Wimmer R.A., Stahl M., Leopoldi A., Garreta E., Hurtado del Pozo C., Prosper F. (2020). Inhibition of SARS-CoV-2 Infections in Engineered Human Tissues Using Clinical-Grade Soluble Human ACE2. Cell.

[52] Adiraju A., Jalasutram A., Al-Hamry A., Talbi M., Wang J., Tegenkamp C., Kanoun O. (2024). Laser-induced fibers and copper phthalocyanine modified laser-induced graphene electrodes for sensitive and selective electrochemical detection of nitrite. RSC Adv..

[53] Wanjari V.P., Duttagupta S.P., Singh S.P. (2023). Dual Linear Range Laser-Induced Graphene-Based Sensor for 4-Nitrophenol Detection in Water. ACS Appl. Nano Mater..

[54] Lobo L.C. (2017). Artificial intelligence and medicine. Rev. Bras. Educ. Med..

[55] Santos M.K., Ferreira Júnior J.R., Wada D.T., Tenório A.P.M., Nogueira-Barbosa M.H., Marques P.M.d.A. (2019). Inteligência artificial, aprendizado de máquina, diagnóstico auxiliado por computador e radiômica: Avanços da imagem rumo à medicina de precisão. Radiol. Bras..

