# Supplementary material for: Electrochemical Immunosensors on Laser-Induced Graphene Platforms for Monitoring of Anti-RBD Antibodies After SARS-CoV-2 Infection
Source: Biosensors (Basel). 2024 Oct 22;14(11):514. doi: 10.3390/bios14110514 (PMC11591629; doi:10.3390/bios14110514)
Supplement: Supplementary file 1 [file biosensors-14-00514-s001.zip › biosensors-3161985-supplementary.pdf]

Table S1. Comparison of SARS-COV-2 detection methods.

Method	Material	Sensitivity	Specificity	Detection limit	Confirmed by	Observations	References
Sofia 2 Flu + SARS Antigen Fluorescent Immunoassay (FIA) kit and analyzer (Quidel Co., San Diego, CA, USA) (Rapid antigen detection)	Oropharyngeal swab	86%	100%	Not available	real-time reverse-transcriptase polymerase chain reaction (rRT-PCR) (GeneXpert GX-XVI instrument; Cepheid, Sunnyvale, CA, USA) (Kappa = 0.8)	Simultaneous detection and differentiation of the nucleocapsid antigens from SARS-CoV-2, influenza A, and influenza B.	[1]
Sofia 2 Flu + SARS Antigen Fluorescent Immunoassay (FIA) kit and analyzer (Quidel Co., San Diego, CA, USA) (Rapid antigen detection)	Nasal	76%	100%	Not available	real-time reverse-transcriptase polymerase chain reaction (rRT-PCR) (GeneXpert GX-XVI instrument; Cepheid, Sunnyvale, CA, USA) (Kappa = 0.68)	Simultaneous detection and differentiation of the nucleocapsid antigens from SARS-CoV-2, influenza A, and influenza B.	[1]
Sofia 2 Flu + SARS Antigen Fluorescent Immunoassay (FIA) kit and analyzer (Quidel Co., San Diego, CA, USA) (Rapid antigen detection)	Saliva	66%	100%	Not available	real-time reverse-transcriptase polymerase chain reaction (rRT-PCR) (GeneXpert GX-XVI instrument; Cepheid, Sunnyvale, CA,	Simultaneous detection and differentiation of the nucleocapsid antigens from SARS-CoV-2, influenza A, and influenza B.	[1]

					USA) (Kappa = 0.57)		
SARS-CoV-2 nucleocapsid (N)-protein produced in Escherichia coli and purified to develop an indirect enzyme-linked immunosorbent assay (ELISA) for the detection of SARS-CoV-2 specific antibodies (Indirect ELISA)	Serum samples	39.1% - first six days 86.4% - second week 89.7% - more than 14 days (number of days after symptom onset + positive PCR test).	99.3%	Not available	RT-PCR	Serum samples with confirmed antibody titers against human immunodeficiency viruses 1/2, parvovirus B19, hepatitis A/B virus, cytomegalovirus, Epstein Barr virus, and herpes simplex virus were tested negative.	[2]
Indirect ELISA for IgG antibodies specific to the SARS-CoV-2 S protein	Serum, plasma, and dried blood spots	100%	98.3%	0.114 BAU/mL	PCR test or samples from COVID-19 vaccine recipients	Sera of patients with confirmed past infections with HIV 1/2, parvovirus B19, hepatitis A/B virus, cytomegalovirus, Epstein–Barr virus, herpes simplex virus, hCoV HKU-1, influenza H1N1, or influenza H5N1.	[3]

ELISA anti-RBD IgG	Serum	90.5*	99.5	Not available	RT-PCR	* > 3 weeks post-symptom onset	[4]
Indirect ELISA for SARS-CoV-2 spike-specific IgG	Serum and plasma	98.1%	99.5%	Not available	In-vitro diagnostics certified Euroimmun anti-SARS-CoV-2 ELISA (IgG).	-----	[5]
Genosensor	Nasopharyngeal swab	Not available	Not available	64fM (4.8pg/ml)	RT-PCR	-----	[6]
Immunosensor	Nasopharyngeal swab and Oropharyngeal swab	Not available	Not available	Receptor CD147: 38.99 ng mL^{-1}. Receptor ACE2: 299.30 ng mL^{-1}	RT-qPCR	-----	[7]
Immunosensor	Nasopharyngeal swab	Not available	Not available	0.25 fg/mL	RT-PCR	-----	[8]
Immunosensor	Nasopharyngeal swab	Not available	Not available	0.8 pg/mL	RT-PCR	-----	[9]
Immunosensor	Saliva	Not available	Not available	Protein S: 19 ng/mL Protein N: 8 ng/mL	qPCR	-----	[10]
Immunosensor	Serum	Not available	Not available	0.032 μg L^{-1}	ELISA and Immunochromatography (rapid test)	-----	This work

Table S1. Detailed comparison of the SARS-CoV-2 detection assays performed in this study with those found in the literature. The different types of samples, RNA degradation methods, RT-qPCR kits used, target genes, detection limits and intra- and inter-assay coefficients of variation are presented. This comparison allows us to evaluate the performance of the methods used in this work in relation to other tests described in

the literature, highlighting the advantages and limitations of each approach. Furthermore, this table presents a comparison between several electrochemical sensors developed to detect SARS-CoV-2, including the sensor proposed in this study. The results obtained demonstrate that the LIG-based sensor has a significantly low detection limit. The performance of the LIG-based sensor can be attributed to the unique properties of graphene, which provide high surface area, excellent electrical conductivity, and functionalization ability. These characteristics make LIGs promising materials for the development of high-performance electrochemical sensors.

References

1. Alonaizan, F.; Alhumaid, J.; Aljindan, R.; Bedi, S.; Dardas, H.; Abdulfattah, D.; Ashour, H.; Alshahrani, M.; Omar, O. Sensitivity and Specificity of Rapid SARS-CoV-2 Antigen Detection Using Different Sampling Methods: A Clinical Unicentral Study. *Int. J. Environ. Res. Public Health* **2022**, *19*, 1–10, doi:10.3390/ijerph19116836.
2. Luo, J.; Brakel, A.; Krizsan, A.; Ludwig, T.; Mötzing, M.; Volke, D.; Lakowa, N.; Grünewald, T.; Lehmann, C.; Wolf, J.; et al. Sensitive and specific serological ELISA for the detection of SARS - CoV - 2 infections. *Virol. J.* **2022**, *19*, 1–10, doi:10.1186/s12985-022-01768-4.
3. Luo, J.; Klett, J.; Gabert, J.; Lipp, T.; Karbach, J.; Jäger, E.; Borte, S.; Hoffmann, R.; Milkovska-Stamenova, S. A Quantitative ELISA to Detect Anti-SARS-CoV-2 Spike IgG Antibodies in Infected Patients and Vaccinated Individuals. *Microorganisms* **2022**, *10*, 1–15, doi:10.3390/microorganisms10091812.
4. Martínez-Barnetche, J.; Carnalla, M.; Gaspar-Castillo, C.; Basto-Abreu, A.; Lizardi, R.; Antonio, R.A.; Martinez, I.L.; Escamilla, A.C.; Ramirez, O.T.; Palomares, L.A.; et al. Comparable diagnostic accuracy of SARS-CoV-2 Spike RBD and N-specific IgG tests to determine pre-vaccination nation-wide baseline seroprevalence in Mexico. *Sci. Rep.* **2022**, *12*, 1–9, doi:10.1038/s41598-022-22146-8.
5. Appeltrath, G.A.; Parreuter, J.; Lindemann, M.; Klump, H.; Karsten, C.B. An efficient ELISA protocol for measurement of SARS-CoV-2 spike-specific IgG in human plasma and serum samples. *MethodsX* **2024**, *12*, 1–7, doi:10.1016/j.mex.2024.102596.
6. Ayankojo, A.G.; Boroznjak, R.; Reut, J.; Opik, A.; Syritski, V. Molecularly imprinted polymer based electrochemical sensor for quantitative detection of SARS-CoV-2 spike protein. *Sensors Actuators, B Chem.* **2022**, *353*, 131160–131167, doi:10.1016/j.snb.2021.131160.
7. Büyüksünetçi, Y.T.; Çitil, B.E.; Tapan, U.; Anık, Ü. Development and application of a SARS-CoV-2 colorimetric biosensor based on the peroxidase-mimic activity of γ-Fe2O3 nanoparticles. *Microchim. Acta* **2021**, *188*, 1–9, doi:10.1007/S00604-021-04989-6/TABLES/3.
8. Ehsan, M.A.; Khan, S.A.; Rehman, A. Screen-printed graphene/carbon electrodes on paper substrates as impedance sensors for detection of coronavirus in nasopharyngeal fluid samples. *Diagnostics* **2021**, *11*, 1–12, doi:10.3390/diagnostics11061030.
9. Eissa, S.; Zourob, M. Development of a low-cost cotton-tipped electrochemical immunosensor for the detection of SARS-CoV-2. *Anal. Chem.* **2021**, *93*, 1826–1833, doi:10.1021/acs.analchem.0c04719.
10. Fabiani, L.; Saroglia, M.; Galatà, G.; De Santis, R.; Fillo, S.; Luca, V.; Faggioni, G.; D'Amore, N.; Regalbuto, E.; Salvatori, P.; et al. Magnetic beads combined with carbon black-based screen-printed electrodes for COVID-19: A reliable and miniaturized electrochemical immunosensor for SARS-CoV-2 detection in saliva. *Biosens. Bioelectron.* **2021**, *171*, 1–12, doi:10.1016/j.bios.2020.112686.
